# An Abstraction-Based Framework for Neural Network Verification

**DOI:** 10.1007/978-3-030-53288-8_3

**Published:** 2020-06-13

**Authors:** Yizhak Yisrael Elboher, Justin Gottschlich, Guy Katz

**Affiliations:** 8 Microsoft Research Lab, Redmond, WA USA; 9 University of Southern California, Los Angeles, CA USA; 10 The Hebrew University of Jerusalem, Jerusalem, Israel; 11 Intel Labs, Santa Clara, USA

## Abstract

Deep neural networks are increasingly being used as controllers for safety-critical systems. Because neural networks are opaque, certifying their correctness is a significant challenge. To address this issue, several neural network verification approaches have recently been proposed. However, these approaches afford limited scalability, and applying them to large networks can be challenging. In this paper, we propose a framework that can enhance neural network verification techniques by using over-approximation to reduce the size of the network—thus making it more amenable to verification. We perform the approximation such that if the property holds for the smaller (abstract) network, it holds for the original as well. The over-approximation may be too coarse, in which case the underlying verification tool might return a spurious counterexample. Under such conditions, we perform counterexample-guided refinement to adjust the approximation, and then repeat the process. Our approach is orthogonal to, and can be integrated with, many existing verification techniques. For evaluation purposes, we integrate it with the recently proposed Marabou framework, and observe a significant improvement in Marabou’s performance. Our experiments demonstrate the great potential of our approach for verifying larger neural networks.

## Introduction

*Machine programming* (MP), the automatic generation of software, is showing early signs of fundamentally transforming the way software is developed [15]. A key ingredient employed by MP is the *deep neural network* (DNN), which has emerged as an effective means to semi-autonomously implement many complex software systems. DNNs are artifacts produced by *machine learning*: a user provides examples of how a system should behave, and a machine learning algorithm generalizes these examples into a DNN capable of correctly handling inputs that it had not seen before. Systems with DNN components have obtained unprecedented results in fields such as image recognition [24], game playing [33], natural language processing [16], computer networks [28], and many others, often surpassing the results obtained by similar systems that have been carefully handcrafted. It seems evident that this trend will increase and intensify, and that DNN components will be deployed in various safety-critical systems [3, 19].

DNNs are appealing in that (in some cases) they are easier to create than handcrafted software, while still achieving excellent results. However, their usage also raises a challenge when it comes to certification. Undesired behavior has been observed in many state-of-the-art DNNs. For example, in many cases slight perturbations to correctly handled inputs can cause severe errors [26, 35]. Because many practices for improving the reliability of hand-crafted code have yet to be successfully applied to DNNs (e.g., code reviews, coding guidelines, etc.), it remains unclear how to overcome the opacity of DNNs, which may limit our ability to certify them before they are deployed.

To mitigate this, the formal methods community has begun developing techniques for the formal verification of DNNs (e.g., [10, 17, 20, 37]). These techniques can automatically prove that a DNN always satisfies a prescribed property. Unfortunately, the DNN verification problem is computationally difficult (e.g., NP-complete, even for simple specifications and networks [20]), and becomes exponentially more difficult as network sizes increase. Thus, despite recent advances in DNN verification techniques, network sizes remain a severely limiting factor.

In this work, we propose a technique by which the scalability of many existing verification techniques can be significantly increased. The idea is to apply the well-established notion of *abstraction and refinement* [6]: replace a network *N* that is to be verified with a much smaller, *abstract* network, $\bar{N}$, and then verify this $\bar{N}$. Because $\bar{N}$ is smaller it can be verified more efficiently; and it is constructed in such a way that if it satisfies the specification, the original network *N* also satisfies it. In the case that $\bar{N}$ does not satisfy the specification, the verification procedure provides a counterexample *x*. This *x* may be a true counterexample demonstrating that the original network *N* violates the specification, or it may be *spurious*. If *x* is spurious, the network $\bar{N}$ is *refined* to make it more accurate (and slightly larger), and then the process is repeated. A particularly useful variant of this approach is to use the spurious *x* to guide the refinement process, so that the refinement step rules out *x* as a counterexample. This variant, known as *counterexample-guided abstraction refinement* (*CEGAR*) [6], has been successfully applied in many verification contexts.

As part of our technique we propose a method for abstracting and refining neural networks. Our basic abstraction step *merges* two neurons into one, thus reducing the overall number of neurons by one. This basic step can be repeated numerous times, significantly reducing the network size. Conversely, refinement is performed by splitting a previously merged neuron in two, increasing the network size but making it more closely resemble the original. A key point is that not all pairs of neurons can be merged, as this could result in a network that is smaller but is not an over-approximation of the original. We resolve this by first transforming the original network into an equivalent network where each node belongs to one of four classes, determined by its edge weights and its effect on the network’s output; merging neurons from the same class can then be done safely. The actual choice of which neurons to merge or split is performed heuristically. We propose and discuss several possible heuristics.

For evaluation purposes, we implemented our approach as a Python framework that wraps the Marabou verification tool [22]. We then used our framework to verify properties of the Airborne Collision Avoidance System (ACAS Xu) set of benchmarks [20]. Our results strongly demonstrate the potential usefulness of abstraction in enhancing existing verification schemes: specifically, in most cases the abstraction-enhanced Marabou significantly outperformed the original. Further, in most cases the properties in question could indeed be shown to hold or not hold for the original DNN by verifying a small, abstract version thereof.

To summarize, our contributions are: (i) we propose a general framework for over-approximating and refining DNNs; (ii) we propose several heuristics for abstraction and refinement, to be used within our general framework; and (iii) we provide an implementation of our technique that integrates with the Marabou verification tool and use it for evaluation. Our code is available online [9].

The rest of this paper is organized as follows. In Sect. 2, we provide a brief background on neural networks and their verification. In Sect. 3, we describe our general framework for abstracting an refining DNNs. In Sect. 4, we discuss how to apply these abstraction and refinement steps as part of a CEGAR procedure, followed by an evaluation in Sect. 5. In Sect. 6, we discuss related work, and we conclude in Sect. 7.

## Background

### Neural Networks

**Fig. 1.**
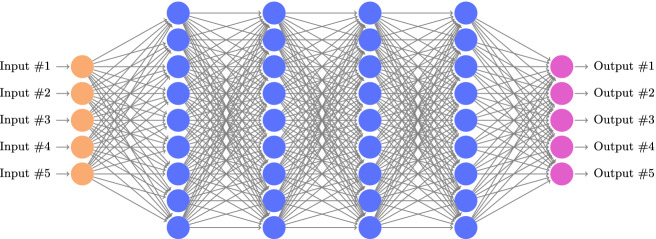
A fully connected, feedforward DNN with 5 input nodes (in orange), 5 output nodes (in purple), and 4 hidden layers containing a total of 36 hidden nodes (in blue). Each edge is associated with a weight value (not depicted). (Color figure online)

A neural network consists of an *input layer*, an *output layer*, and one or more intermediate layers called *hidden layers*. Each layer is a collection of nodes, called *neurons*. Each neuron is connected to other neurons by one or more directed edges. In a feedforward neural network, the neurons in the first layer receive input data that sets their initial values. The remaining neurons calculate their values using the weighted values of the neurons that they are connected to through edges from the preceding layer (see Fig. 1). The output layer provides the resulting value of the DNN for a given input.

There are many types of DNNs, which may differ in the way their neuron values are computed. Typically, a neuron is evaluated by first computing a weighted sum of the preceding layer’s neuron values according to the edge weights, and then applying an activation function to this weighted sum [13]. We focus here on the Rectified Linear Unit (ReLU) activation function [29], given as $\text {ReLU} {}{}(x) = \max {}(0, x)$. Thus, if the weighted sum computation yields a positive value, it is kept; and otherwise, it is replaced by zero.

More formally, given a DNN *N*, we use *n* to denote the number of layers of *N*. We denote the number of nodes of layer *i* by $s_i$. Layers 1 and *n* are the input and output layers, respectively. Layers $2,\ldots ,n-1$ are the hidden layers. We denote the value of the *j*-th node of layer *i* by $v_{i,j}$, and denote the column vector $[v_{i,1},\ldots ,v_{i,s_i}]^T$ as $V_i$.

Evaluating *N* is performed by calculating $$V_n$$ for a given input assignment $$V_1$$. This is done by sequentially computing $$V_i$$ for $$i=2,3,\ldots ,n$$, each time using the values of $$V_{i-1}$$ to compute weighted sums, and then applying the ReLU activation functions. Specifically, layer *i* (for $$i>1$$) is associated with a weight matrix $$W_i$$ of size $$s_{i}\times s_{i-1}$$ and a bias vector $$B_i$$ of size $$ s_i$$. If *i* is a hidden layer, its values are given by $$ V_i = \text {ReLU} {}{}(W_i V_{i-1} + B_i), $$ where the ReLUs are applied element-wise; and the output layer is given by $$V_n = W_nV_{n-1}+B_n$$ (ReLUs are not applied). Without loss of generality, in the rest of the paper we assume that all bias values are 0, and can be ignored. This rule is applied repeatedly once for each layer, until $$V_n$$ is eventually computed.

We will sometimes use the notation $w(v_{i,j},v_{i+1,k})$ to refer to the entry of $W_{i+1}$ that represents the weight of the edge between neuron *j* of layer *i* and neuron *k* of layer $i+1$. We will also refer to such an edge as an *outgoing edge* for $v_{i,j}$, and as an *incoming edge* for $v_{i+1,k}$.

As part of our abstraction framework, we will sometimes need to consider a *suffix* of a DNN, in which the first layers of the DNN are omitted. For $1<i<n$, we use $N^{[i]}$ to denote the DNN comprised of layers $i, i+1,\ldots ,n$ of the original network. The sizes and weights of the remaining layers are unchanged, and layer *i* of *N* is treated as the input layer of $N^{[i]}$.

**Fig. 2.**
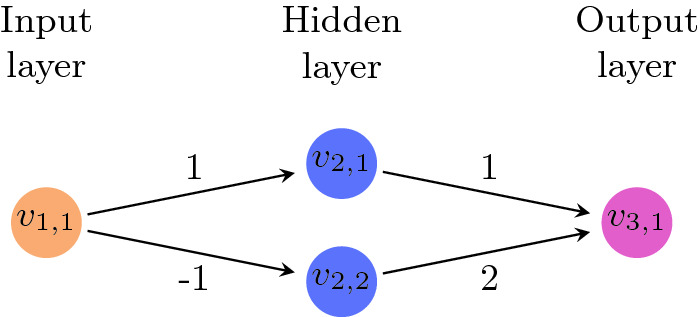
A simple feedforward neural network.

Figure 2 depicts a small neural network. The network has $n=3$ layers, of sizes $s_1=1, s_2=2$ and $s_3=1$. Its weights are $w(v_{1,1},v_{2,1})=1$, $w(v_{1,1},v_{2,2})=-1$, $w(v_{2,1},v_{3,1})=1$ and $w(v_{2,2},v_{3,1})=2$. For input $v_{1,1}=3$, node $v_{2,1}$ evaluates to 3 and node $v_{2,2}$ evaluates to 0, due to the ReLU activation function. The output node $v_{3,1}$ then evaluates to 3.

### Neural Network Verification

DNN verification amounts to answering the following question: given a DNN *N*, which maps input vector *x* to output vector *y*, and predicates *P* and *Q*, does there exist an input $x_0$ such that $P(x_0)$ and $Q(N(x_0))$ both hold? In other words, the verification process determines whether there exists a particular input that meets the input criterion *P*, and that is mapped to an output that meets the output criterion *Q*. We refer to $\langle N, P, Q\rangle$ as the *verification query*. As is usual in verification, *Q* represents the *negation* of the desired property. Thus, if the query is *unsatisfiable* (UNSAT), the property holds; and if it is *satisfiable* (SAT), then $x_0$ constitutes a counterexample to the property in question.

**Fig. 3.**
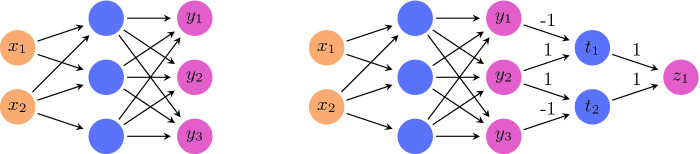
Reducing a complex property to the $y>0$ form. For the network on the left hand side, suppose we wish to examine the property $y_2>y_1 \vee y_2 > y_3$, which is a property that involves multiple outputs and includes a disjunction. We do this (right hand side network) by adding two neurons, $t_1$ and $t_2$, such that $t_1=\text {ReLU} {}{}(y_2-y_1)$ and $t_2=\text {ReLU} {}{}(y_2-y_3)$. Thus, $t_1>0$ if and only if the first disjunct, $y_2>y_1$, holds; and $t_2>0$ if and only if the second disjunct, $y_2>y_3$, holds. Finally, we add a neuron $z_1$ such that $z_1=t_1 + t_2$. It holds that $z_1>0$ if and only if $t_1>0\vee t_2>0$. Thus, we have reduced the complex property into an equivalent property in the desired form.

Different verification approaches may differ in (i) the kinds of neural networks they allow (specifically, the kinds of activation functions in use); (ii) the kinds of input properties; and (iii) the kinds of output properties. For simplicity, we focus on networks that employ the ReLU activation function. In addition, our input properties will be conjunctions of linear constraints on the input values. Finally, we will assume that our networks have a single output node *y*, and that the output property is $y > c$ for a given constant *c*. We stress that these restrictions are for the sake of simplicity. Many properties of interest, including those with arbitrary Boolean structure and involving multiple neurons, can be reduced into the above single-output setting by adding a few neurons that encode the Boolean structure [20, 32]; see Fig. 3 for an example. The number of neurons to be added is typically negligible when compared to the size of the DNN. In particular, this is true for the ACAS Xu family of benchmarks [20], and also for adversarial robustness queries that use the $L_\infty$ or the $L_1$ norm as a distance metric [5, 14, 21]. Additionally, other piecewise-linear activation functions, such as max-pooling layers, can also be encoded using ReLU s [5].

Several techniques have been proposed for solving the aforementioned verification problem in recent years (Sect. 6 includes a brief overview). Our abstraction technique is designed to be compatible with most of these techniques, by simplifying the network being verified, as we describe next.

## Network Abstraction and Refinement

Because the complexity of verifying a neural network is strongly connected to its size [20], our goal is to transform a verification query $\varphi _1 = \langle N, P, Q\rangle$ into query $\varphi _2=\langle \bar{N}, P, Q\rangle$, such that the abstract network $\bar{N}$ is significantly smaller than *N* (notice that properties *P* and *Q* remain unchanged). We will construct $\bar{N}$ so that it is an over-approximation of *N*, meaning that if $\varphi _2$ is UNSAT then $\varphi _1$ is also UNSAT. More specifically, since our DNNs have a single output, we can regard *N*(*x*) and $\bar{N}(x)$ as real values for every input *x*. To guarantee that $\varphi _2$ over-approximates $\varphi _1$, we will make sure that for every *x*, $N(x)\le \bar{N}(x)$; and thus, $\bar{N}(x)\le c \implies N(x)\le c$. Because our output properties always have the form $N(x)>c$, it is indeed the case that if $\varphi _2$ is UNSAT, i.e. $\bar{N}(x)\le c$ for all *x*, then $N(x)\le c$ for all *x* and so $\varphi _1$ is also UNSAT. We now propose a framework for generating various $\bar{N}$s with this property.

### Abstraction

We seek to define an abstraction operator that removes a single neuron from the network, by merging it with another neuron. To do this, we will first transform *N* into an equivalent network, whose neurons have properties that will facilitate their merging. Equivalent here means that for every input vector, both networks produce the exact same output. First, each hidden neuron $v_{i,j}$ of our transformed network will be classified as either a pos neuron or a neg neuron. A neuron is pos if all the weights on its outgoing edges are positive, and is neg if all those weights are negative. Second, orthogonally to the pos/neg classification, each hidden neuron will also be classified as either an inc neuron or a dec neuron. $v_{i,j}$ is an inc neuron of *N* if, when we look at $N^{[i]}$ (where $v_{i,j}$ is an input neuron), increasing the value of $v_{i,j}$ increases the value of the network’s output. Formally, $v_{i,j}$ is inc if for every two input vectors $x_1$ and $x_2$ where $x_1[k]=x_2[k]$ for $k\ne j$ and $x_1[j]>x_2[j]$, it holds that $N^{[i]}(x_1) > N^{[i]}(x_2)$. A dec neuron is defined symmetrically, so that *decreasing* the value of *x*[*j*] *increases* the output. We first describe this transformation (an illustration of which appears in Fig. 4), and later we explain how it fits into our abstraction framework.

Our first step is to transform *N* into a new network, $N'$, in which every hidden neuron is classified as pos or neg. This transformation is done by replacing each hidden neuron $v_{i_j}$ with two neurons, $v^+_{i,j}$ and $v^-_{i,j}$, which are respectively pos and neg. Both $v^+_{i,j}$ an $v^-_{i,j}$ retain a copy of all incoming edges of the original $v_{i,j}$; however, $v^+_{i,j}$ retains just the outgoing edges with positive weights, and $v^-_{i,j}$ retains just those with negative weights. Outgoing edges with negative weights are removed from $v^+_{i,j}$ by setting their weights to 0, and the same is done for outgoing edges with positive weights for $v^-_{i,j}$. Formally, for every neuron $v_{i-1,p}$,

$$ w'(v_{i-1,p},v^+_{i,j}) = w(v_{i-1,p},v_{i,j}), \qquad w'(v_{i-1,p},v^-_{i,j}) = w(v_{i-1,p},v_{i,j}) $$

where $w'$ represents the weights in the new network $N'$. Also, for every neuron $v_{i+1,q}$

$$ w'(v^+_{i,j},v_{i+1,q}) = {\left\{ \begin{array}{ll} w(v_{i,j},v_{i+1,q}) &{} w(v_{i,j},v_{i+1,q})\ge 0 \\ 0 &{} \text {otherwise} \end{array}\right. } $$

and

$$ w'(v^-_{i,j},v_{i+1,q}) = {\left\{ \begin{array}{ll} w(v_{i,j},v_{i+1,q}) &{} w(v_{i,j},v_{i+1,q})\le 0 \\ 0 &{} \text {otherwise} \end{array}\right. } $$

(see Fig. 4). This operation is performed once for every hidden neuron of *N*, resulting in a network $N'$ that is roughly double the size of *N*. Observe that $N'$ is indeed equivalent to *N*, i.e. their outputs are always identical.

**Fig. 4.**
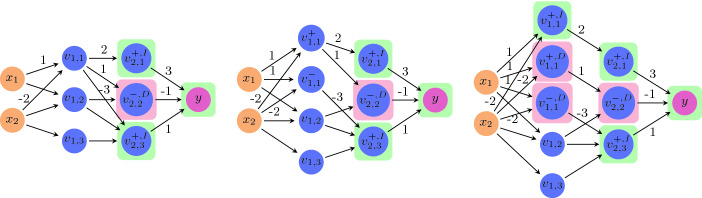
Classifying neurons as pos/neg and inc/dec. In the initial network (left), the neurons of the second hidden layer are already classified: $^+$ and $^-$ superscripts indicate pos and neg neurons, respectively; the $^I$ superscript and green background indicate inc, and the $^D$ superscript and red background indicate dec. Classifying node $v_{1,1}$ is done by first splitting it into two nodes $v^+_{1,1}$ and $v^-_{1,1}$ (middle). Both nodes have identical incoming edges, but the outgoing edges of $v_{1,1}$ are partitioned between them, according to the sign of each edge’s weight. In the last network (right), $v^+_{1,1}$ is split once more, into an inc node with outgoing edges only to other inc nodes, and a dec node with outgoing edges only to other dec nodes. Node $v_{1,1}$ is thus transformed into three nodes, each of which can finally be classified as inc or dec. Notice that in the worst case, each node is split into four nodes, although for $v_{1,1}$ three nodes were enough.

Our second step is to alter $N'$ further, into a new network $N''$, where every hidden neuron is either inc or dec (in addition to already being pos or neg). Generating $N''$ from $N'$ is performed by traversing the layers of $N'$ backwards, each time handling a single layer and possibly doubling its number of neurons:

- Initial step: the output layer has a single neuron, *y*. This neuron is an inc node, because increasing its value will increase the network’s output value.
- Iterative step: observe layer *i*, and suppose the nodes of layer $i+1$ have already been partitioned into inc and dec nodes. Observe a neuron $v^+_{i,j}$ in layer *i* which is marked pos (the case for neg is symmetrical). We replace $v^+_{i,j}$ with two neurons $v^{+,I}_{i,j}$ and $v^{+,D}_{i,j}$, which are inc and dec, respectively. Both new neurons retain a copy of all incoming edges of $v^{+}_{i,j}$; however, $v^{+,I}_{i,j}$ retains only outgoing edges that lead to inc nodes, and $v^{+,D}_{i,j}$ retains only outgoing edges that lead to dec nodes. Thus, for every $v_{i-1,p}$ and $v_{i+1,q}$,
  $$ w''(v_{i-1,p},v^{+,I}_{i,j}) = w'(v_{i-1,p},v^+_{i,j}), \qquad w''(v_{i-1,p},v^{+,D}_{i,j}) = w'(v_{i-1,p},v^+_{i,j}) $$
  $$ w''(v^{+,I}_{i,j},v_{i+1,q}) = {\left\{ \begin{array}{ll} w'(v^+_{i,j},v_{i+1,q}) &{} \text {if } v_{i+1,q} \text { is }\texttt {inc}{} \\ 0 &{} \text {otherwise} \end{array}\right. } $$
  $$ w''(v^{+,D}_{i,j},v_{i+1,q}) = {\left\{ \begin{array}{ll} w'(v^+_{i,j},v_{i+1,q}) &{} \text {if } v_{i+1,q} \text { is }\texttt {dec}{} \\ 0 &{} \text {otherwise} \end{array}\right. } $$
  where $w''$ represents the weights in the new network $N''$. We perform this step for each neuron in layer *i*, resulting in neurons that are each classified as either inc or dec.

To understand the intuition behind this classification, recall that by our assumption all hidden nodes use the ReLU activation function, which is monotonically increasing. Because $v^{+}_{i,j}$ is pos, all its outgoing edges have positive weights, and so if its assignment was to increase (decrease), the assignments of all nodes to which it is connected in the following layer would also increase (decrease). Thus, we split $v^{+}_{i,j}$ in two, and make sure one copy, $v^{+,I}_{i,j}$, is only connected to nodes that need to increase (inc nodes), and that the other copy, $v^{+,D}_{i,j}$, is only connected to nodes that need to decrease (dec nodes). This ensures that $v^{+,I}_{i,j}$ is itself inc, and that $v^{+,D}_{i,j}$ is dec. Also, both $v^{+,I}_{i,j}$ and $v^{+,D}_{i,j}$ remain pos nodes, because their outgoing edges all have positive weights.

When this procedure terminates, $N''$ is equivalent to $N'$, and so also to *N*; and $N''$ is roughly double the size of $N'$, and roughly four times the size of *N*. Both transformation steps are only performed for hidden neurons, whereas the input and output neurons remain unchanged. This is summarized by the following lemma:

#### Lemma 1

Any DNN *N* can be transformed into an equivalent network $N''$ where each hidden neuron is pos or neg, and also inc or dec, by increasing its number of neurons by a factor of at most 4.

Using Lemma 1, we can assume without loss of generality that the DNN nodes in our input query $\varphi _1$ are each marked as pos/neg and as inc/dec. We are now ready to construct the over-approximation network $\bar{N}$. We do this by specifying an abstract operator that merges a pair of neurons in the network (thus reducing network size by one), and can be applied multiple times. The only restrictions are that the two neurons being merged need to be from the same hidden layer, and must share the same pos/neg and inc/dec attributes. Consequently, applying abstract to saturation will result in a network with at most 4 neurons in each hidden layer, which over-approximates the original network. This, of course, would be an immense reduction in the number of neurons for most reasonable input networks.

The abstract operator’s behavior depends on the attributes of the neurons being merged. For simplicity, we will focus on the $\langle \texttt {pos}{},\texttt {inc}{}\rangle$ case. Let $v_{i,j}$, $v_{i,k}$ be two hidden neurons of layer *i*, both classified as $\langle \texttt {pos}{},\texttt {inc}{}\rangle$. Because layer *i* is hidden, we know that layers $i+1$ and $i-1$ are defined. Let $v_{i-1,p}$ and $v_{i+1,q}$ denote arbitrary neurons in the preceding and succeeding layer, respectively. We construct a network $\bar{N}$ that is identical to *N*, except that: (i) nodes $v_{i,j}$ and $v_{i,k}$ are removed and replaced with a new single node, $v_{i,t}$; and (ii) all edges that touched nodes $v_{i,j}$ or $v_{i,k}$ are removed, and other edges are untouched. Finally, we add new incoming and outgoing edges for the new node $v_{i,t}$ as follows:

- Incoming edges: $$ \bar{w}(v_{i-1,p},v_{i,t}) = \max \{w(v_{i-1,p},v_{i,j}), w(v_{i-1,p},v_{i,k})\} $$
- Outgoing edges: $$ \bar{w}(v_{i,t},v_{i+1,q}) = w(v_{i,j},v_{i+1,q}) + w(v_{i,k},v_{i+1,q}) $$

where $\bar{w}$ represents the weights in the new network $\bar{N}$. An illustrative example appears in Fig. 5. Intuitively, this definition of abstract seeks to ensure that the new node $v_{i,t}$ always contributes more to the network’s output than the two original nodes $v_{i,j}$ and $v_{i,k}$—so that the new network produces a larger output than the original for every input. By the way we defined the incoming edges of the new neuron $v_{i,t}$, we are guaranteed that for every input *x* passed into both *N* and $\bar{N}$, the value assigned to $v_{i,t}$ in $\bar{N}$ is greater than the values assigned to both $v_{i,j}$ and $v_{i,k}$ in the original network. This works to our advantage, because $v_{i,j}$ and $v_{i,k}$ were both inc—so increasing their values increases the output value. By our definition of the outgoing edges, the values of any inc nodes in layer $i+1$ increase in $\bar{N}$ compared to *N*, and those of any dec nodes decrease. By definition, this means that the network’s overall output increases.

The abstraction operation for the $\langle \texttt {neg}{},\texttt {inc}{}\rangle$ case is identical to the one described above. For the remaining two cases, i.e. $\langle \texttt {pos}{},\texttt {dec}{}\rangle$ and $\langle \texttt {neg}{},\texttt {dec}{}\rangle$, the $\max$ operator in the definition is replaced with a $\min$ operator.

The next lemma (proof omitted due to lack of space) justifies the use of our abstraction step, and can be applied once per each application of abstract:

#### Lemma 2

Let $\bar{N}$ be derived from *N* by a single application of abstract. For every *x*, it holds that $\bar{N}(x)\ge N(x)$.

**Fig. 5.**
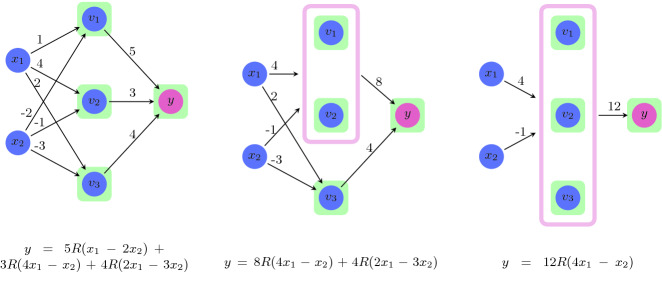
Using abstract to merge $\langle \texttt {pos}{},\texttt {inc}{}\rangle$ nodes. Initially (left), the three nodes $v_1,v_2$ and $v_3$ are separate. Next (middle), abstract merges $v_1$ and $v_2$ into a single node. For the edge between $x_1$ and the new abstract node we pick the weight 4, which is the maximal weight among edges from $x_1$ to $v_1$ and $v_2$. Likewise, the edge between $x_2$ and the abstract node has weight $-1$. The outgoing edge from the abstract node to *y* has weight 8, which is the sum of the weights of edges from $v_1$ and $v_2$ to *y*. Next, abstract is applied again to merge $v_3$ with the abstract node, and the weights are adjusted accordingly (right). With every abstraction, the value of *y* (given as a formula at the bottom of each DNN, where *R* represents the ReLU operator) increases. For example, to see that $12R(4x_1-x_2)\ge 8R(4x_1-x_2) + 4R(2x_1-3x_2)$, it is enough to see that $4R(4x_1-x_2)\ge 4R(2x_1-3x_2)$, which holds because ReLU is a monotonically increasing function and $x_1$ and $x_2$ are non-negative (being, themselves, the output of ReLU nodes).

### Refinement

The aforementioned abstract operator reduces network size by merging neurons, but at the cost of accuracy: whereas for some input $x_0$ the original network returns $N(x_0)=3$, the over-approximation network $\bar{N}$ created by abstract might return $\bar{N}(x_0)=5$. If our goal is prove that it is never the case that $N(x)>10$, this over-approximation may be adequate: we can prove that always $\bar{N}(x)\le 10$, and this will be enough. However, if our goal is to prove that it is never the case that $N(x)>4$, the over-approximation is inadequate: it is possible that the property holds for *N*, but because $\bar{N}(x_0)=5>4$, our verification procedure will return $x_0$ as a *spurious counterexample* (a counterexample for $\bar{N}$ that is not a counterexample for *N*). In order to handle this situation, we define a *refinement operator*, refine, that is the inverse of abstract: it transforms $\bar{N}$ into yet another over-approximation, $\bar{N}'$, with the property that for every *x*, $N(x)\le \bar{N}'(x)\le \bar{N}(x)$. If $\bar{N}'(x_0)=3.5$, it might be a suitable over-approximation for showing that never $N(x)>4$. In this section we define the refine operator, and in Sect. 4 we explain how to use abstract and refine as part of a CEGAR-based verification scheme.

Recall that abstract merges together a couple of neurons that share the same attributes. After a series of applications of abstract, each hidden layer *i* of the resulting network can be regarded as a partitioning of hidden layer *i* of the original network, where each partition contains original, *concrete* neurons that share the same attributes. In the abstract network, each partition is represented by a single, *abstract* neuron. The weights on the incoming and outgoing edges of this abstract neuron are determined according to the definition of the abstract operator. For example, in the case of an abstract neuron $\bar{v}$ that represents a set of concrete neurons $\{v_1,\ldots ,v_n\}$ all with attributes $\langle \texttt {pos}{},\texttt {inc}{}\rangle$, the weight of each incoming edge to $\bar{v}$ is given by

$$ \bar{w}(u,v) = \max (w(u,v_1),\ldots ,w(u,v_n)) $$

where *u* represents a neuron that has not been abstracted yet, and *w* is the weight function of the original network. The key point here is that the order of abstract operations that merged $v_1,\ldots ,v_n$ does not matter—but rather, only the fact that they are now grouped together determines the abstract network’s weights. The following corollary, which is a direct result of Lemma 2, establishes this connection between sequences of abstract applications and partitions:

#### Corollary 1

Let *N* be a DNN where each hidden neuron is labeled as pos/neg and inc/dec, and let $\mathcal {P}$ be a partitioning of the hidden neurons of *N*, that only groups together hidden neurons from the same layer that share the same labels. Then *N* and $\mathcal {P}$ give rise to an abstract neural network $\bar{N}$, which is obtained by performing a series of abstract operations that group together neurons according to the partitions of $\mathcal {P}$. This $\bar{N}$ is an over-approximation of *N*.

We now define a refine operation that is, in a sense, the inverse of abstract. refine takes as input a DNN $\bar{N}$ that was generated from *N* via a sequence of abstract operations, and splits a neuron from $\bar{N}$ in two. Formally, the operator receives the original network *N*, the partitioning $\mathcal {P}$, and a finer partition $\mathcal {P}'$ that is obtained from $\mathcal {P}$ by splitting a single class in two. The operator then returns a new abstract network, $\bar{N}'$, that is the abstraction of *N* according to $\mathcal {P}'$.

Due to Corollary 1, and because $\bar{N}$ returned by refine corresponds to a partition $\mathcal {P}'$ of the hidden neurons of *N*, it is straightforward to show that $\bar{N}$ is indeed an over-approximation of *N*. The other useful property that we require is the following:

#### Lemma 3

Let $\bar{N}$ be an abstraction of *N*, and let $\bar{N}'$ be a network obtained from $\bar{N}$ by applying a single refine step. Then for every input *x* it holds that $\bar{N}(x)\ge \bar{N}'(x)\ge N(x)$.

The second part of the inequality, $\bar{N}'(x)\ge N(x)$ holds because $\bar{N}'$ is an over-approximation of *N* (Corollary 1). The first part of the inequality, $\bar{N}(x)\ge \bar{N}'(x)$, follows from the fact that $\bar{N}(x)$ can be obtained from $\bar{N}'(x)$ by a single application of abstract.

In practice, in order to support the refinement of an abstract DNN, we maintain the current partitioning, i.e. the mapping from concrete neurons to the abstract neurons that represent them. Then, when an abstract neuron is selected for refinement (according to some heuristic, such as the one we propose in Sect. 4), we adjust the mapping and use it to compute the weights of the edges that touch the affected neuron.

## A CEGAR-Based Approach

In Sect. 3 we defined the abstract operator that reduces network size at the cost of reducing network accuracy, and its inverse refine operator that increases network size and restores accuracy. Together with a black-box verification procedure *Verify* that can dispatch queries of the form $\varphi = \langle N, P, Q\rangle$, these components now allow us to design an abstraction-refinement algorithm for DNN verification, given as Algorithm 1 (we assume that all hidden neurons in the input network have already been marked pos/neg and inc/dec).
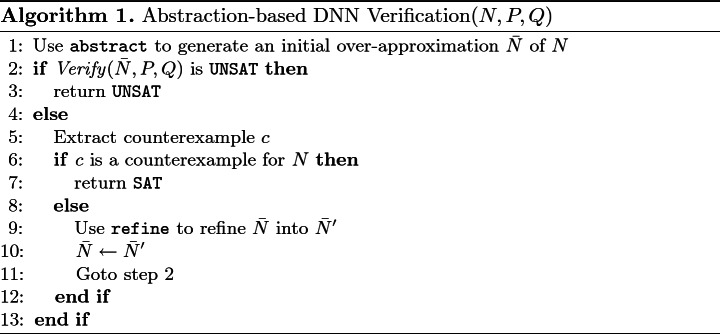


Because $\bar{N}$ is obtained via applications of abstract and refine, the soundness of the underlying *Verify* procedure, together with Lemmas 2 and 3, guarantees the soundness of Algorithm 1. Further, the algorithm always terminates: this is the case because all the abstract steps are performed first, followed by a sequence of refine steps. Because no additional abstract operations are performed beyond Step 1, after finitely many refine steps $\bar{N}$ will become identical to *N*, at which point no spurious counterexample will be found, and the algorithm will terminate with either SAT or UNSAT. Of course, termination is only guaranteed when the underlying *Verify* procedure is guaranteed to terminate.

There are two steps in the algorithm that we intentionally left ambiguous: Step 1, where the initial over-approximation is computed, and Step 9, where the current abstraction is refined due to the discovery of a spurious counterexample. The motivation was to make Algorithm 1 general, and allow it to be customized by plugging in different heuristics for performing Steps 1 and 9, which may depend on the problem at hand. Below we propose a few such heuristics.

### Generating an Initial Abstraction

The most naïve way to generate the initial abstraction is to apply the abstract operator to saturation. As previously discussed, abstract can merge together any pair of hidden neurons from a given layer that share the same attributes. Since there are four possible attribute combinations, this will result in each hidden layer of the network having four neurons or fewer. This method, which we refer to as *abstraction to saturation*, produces the smallest abstract networks possible. The downside is that, in some case, these networks might be too coarse, and might require multiple rounds of refinement before a SAT or UNSAT answer can be reached.

A different heuristic for producing abstractions that may require fewer refinement steps is as follows. First, we select a finite set of input points, $X=\{x_1,\ldots ,x_n\}$, all of which satisfy the input property *P*. These points can be generated randomly, or according to some coverage criterion of the input space. The points of *X* are then used as indicators in estimating when the abstraction has become too coarse: after every abstraction step, we check whether the property still holds for $x_1,\ldots ,x_n$, and stop abstracting if this is not the case. The exact technique, which we refer to as *indicator-guided abstraction*, appears in Algorithm 2, which is used to perform Step 1 of Algorithm 1.
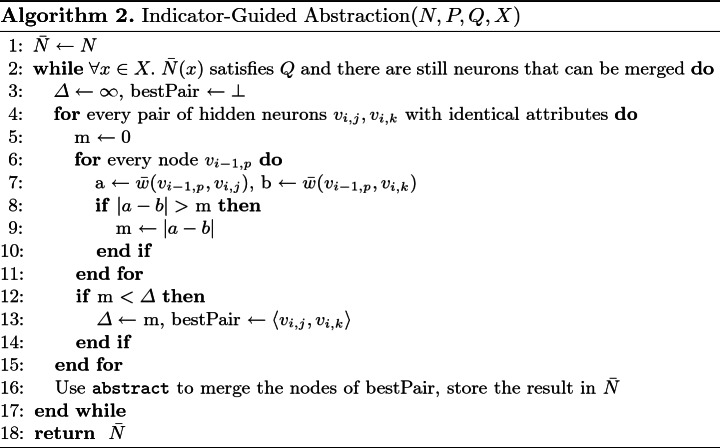


Another point that is addressed by Algorithm 2, besides how many rounds of abstraction should be performed, is which pair of neurons should be merged in every application of abstract. This, too, is determined heuristically. Since any pair of neurons that we pick will result in the same reduction in network size, our strategy is to prefer neurons that will result in a more accurate approximation. Inaccuracies are caused by the $\max {}$ and $\min {}$ operators within the abstract operator: e.g., in the case of $\max {}$, every pair of incoming edges with weights *a*, *b* are replaced by a single edge with weight $\max {}(a,b)$. Our strategy here is to merge the pair of neurons for which the *maximal* value of $|a-b|$ (over all incoming edges with weights *a* and *b*) is *minimal*. Intuitively, this leads to $\max {}(a,b)$ being close to both *a* and *b*—which, in turn, leads to an over-approximation network that is smaller than the original, but is close to it weight-wise. We point out that although repeatedly exploring all pairs (line 4) may appear costly, in our experiments the time cost of this step was negligible compared to that of the verification queries that followed. Still, if this step happens to become a bottleneck, it is possible to adjust the algorithm to heuristically sample just some of the pairs, and pick the best pair among those considered—without harming the algorithm’s soundness.

As a small example, consider the network depicted on the left hand side of Fig. 5. This network has three pairs of neurons that can be merged using abstract (any subset of $\{v_1,v_2,v_3\}$). Consider the pair $v_1,v_2$: the maximal value of $|a-b|$ for these neurons is $\max {}(|1-4)|,|(-2)-(-1)|)=3$. For pair $v_1,v_3$, the maximal value is 1; and for pair $v_2,v_3$ the maximal value is 2. According to the strategy described in Algorithm 2, we would first choose to apply abstract on the pair with the minimal maximal value, i.e. on the pair $v_1, v_3$.

### Performing the Refinement Step

A refinement step is performed when a spurious counterexample *x* has been found, indicating that the abstract network is too coarse. In other words, our abstraction steps, and specifically the $\max {}$ and $\min {}$ operators that were used to select edge weights for the abstract neurons, have resulted in the abstract network’s output being too great for input *x*, and we now need to reduce it. Thus, our refinement strategies are aimed at applying refine in a way that will result in a significant reduction to the abstract network’s output. We note that there may be multiple options for applying refine, on different nodes, such that any of them would remove the spurious counterexample *x* from the abstract network. In addition, it is not guaranteed that it is possible to remove *x* with a single application of refine, and multiple consecutive applications may be required.

One heuristic approach for refinement follows the well-studied notion of counterexample-guided abstraction refinement [6]. Specifically, we leverage the spurious counterexample *x* in order to identify a concrete neuron *v*, which is currently mapped into an abstract neuron $\bar{v}$, such that splitting *v* away from $\bar{v}$ might rule out counterexample *x*. To do this, we evaluate the original network on *x* and compute the value of *v* (we denote this value by *v*(*x*)), and then do the same for $\bar{v}$ in the abstract network (value denoted $\bar{v}(x)$). Intuitively, a neuron pair $\langle v, \bar{v}\rangle$ for which the difference $|v(x) - \bar{v}(x)|$ is significant makes a good candidate for a refinement operation that will split *v* away from $\bar{v}$.

In addition to considering *v*(*x*) and $\bar{v}(x)$, we propose to also consider the weights of the incoming edges of *v* and $\bar{v}$. When these weights differ significantly, this could indicate that $\bar{v}$ is too coarse an approximation for *v*, and should be refined. We argue that by combining these two criteria—edge weight difference between *v* and $\bar{v}$, which is a property of the current abstraction, together with the difference between *v*(*x*) and $\bar{v}(x)$, which is a property of the specific input *x*, we can identify abstract neurons that have contributed significantly to *x* being a spurious counterexample.

The refinement heuristic is formally defined in Algorithm 3. The algorithm traverses the original neurons, looks for the edge weight times assignment value that has changed the most as a result of the current abstraction, and then performs refinement on the neuron at the end of that edge. As was the case with Algorithm 2, if considering all possible nodes turns out to be too costly, it is possible to adjust the algorithm to explore only some of the nodes, and pick the best one among those considered—without jeopardizing the algorithm’s soundness.
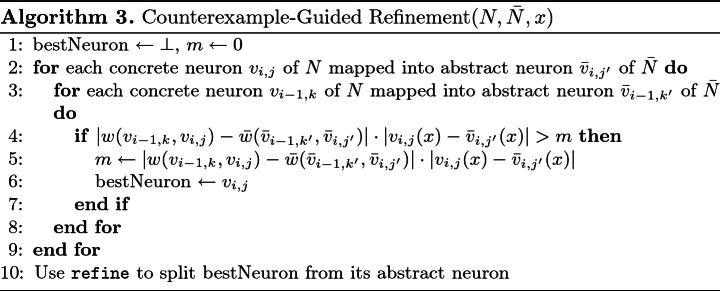


As an example, let us use Algorithm 3 to choose a refinement step for the right hand side network of Fig. 5, for a spurious counterexample $\langle x_1,x_2\rangle = \langle 1, 0\rangle$. For this input, the original neurons’ evaluation is $v_1=1, v_2=4$ and $v_3=2$, whereas the abstract neuron that represents them evaluates to 4. Suppose $v_1$ is considered first. In the abstract network, $\bar{w}(x_1,\bar{v_1})=4$ and $\bar{w}(x_2,\bar{v_1})=-1$; whereas in the original network, $w(x_1,v_1)=1$ and $w(x_2,v_1)=-2$. Thus, the largest value *m* computed for $v_1$ is $|w(x_1,v_1) - \bar{w}(x_1,\bar{v_1})|\cdot |4 -1| = 3\cdot 3 = 9$. This value of *m* is larger than the one computed for $v_2$ (0) and for $v_3$ (4), and so $v_1$ is selected for the refinement step. After this step is performed, $v_2$ and $v_3$ are still mapped to a single abstract neuron, whereas $v_1$ is mapped to a separate neuron in the abstract network.

## Implementation and Evaluation

Our implementation of the abstraction-refinement framework includes modules that read a DNN in the NNet format [19] and a property to be verified, create an initial abstract DNN as described in Sect. 4, invoke a black-box verification engine, and perform refinement as described in Sect. 4. The process terminates when the underlying engine returns either UNSAT, or an assignment that is a true counterexample for the original network. For experimentation purposes, we integrated our framework with the Marabou DNN verification engine [22]. Our implementation and benchmarks are publicly available online [9].

**Fig. 6.**
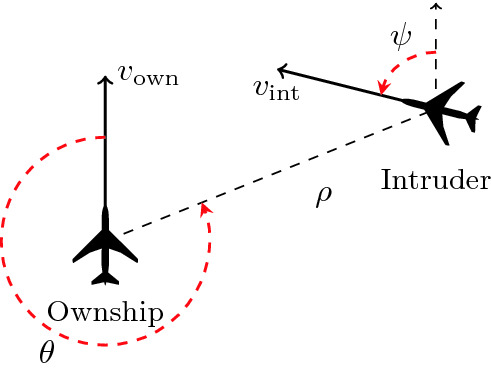
(From [20]) An illustration of the sensor readings passed as input to the ACAS Xu DNNs.

Our experiments included verifying several properties of the 45 ACAS Xu DNNs for airborne collision avoidance [19, 20]. ACAS Xu is a system designed to produce horizontal turning advisories for an unmanned aircraft (the *ownship*), with the purpose of preventing a collision with another nearby aircraft (the *intruder*). The ACAS Xu system receive as input sensor readings, indicating the location of the intruder relative to the ownship, the speeds of the two aircraft, and their directions (see Fig. 6). Based on these readings, it selects one of 45 DNNs, to which the readings are then passed as input. The selected DNN then assigns scores to five output neurons, each representing a possible turning advisory: strong left, weak left, strong right, weak right, or clear-of-conflict (the latter indicating that it is safe to continue along the current trajectory). The neuron with the *lowest* score represents the selected advisory. We verified several properties of these DNNs based on the list of properties that appeared in [20]—specifically focusing on properties that ensure that the DNNs always advise clear-of-conflict for distant intruders, and that they are robust to (i.e., do not change their advisories in the presence of) small input perturbations.

Each of the ACAS Xu DNNs has 300 hidden nodes spread across 6 hidden layers, leading to 1200 neurons when the transformation from Sect. 3.1 is applied. In our experiments we set out to check whether the abstraction-based approach could indeed prove properties of the ACAS Xu networks on abstract networks that had significantly fewer neurons than the original ones. In addition, we wished to compare the proposed approaches for generating initial abstractions (the abstraction to saturation approach versus the indicator-guided abstraction described in Algorithm 2), in order to identify an optimal configuration for our tool. Finally, once the optimal configuration has been identified, we used it to compare our tool’s performance to that of vanilla Marabou. The results are described next.

**Fig. 7.**
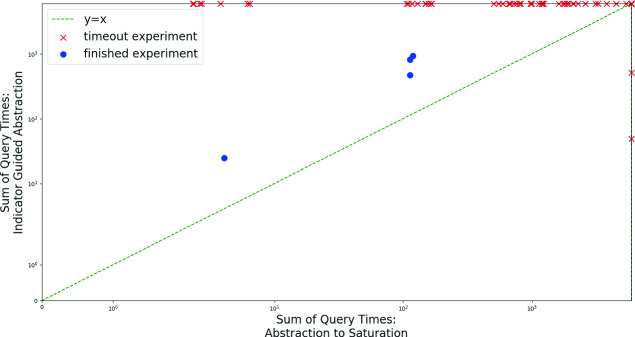
Generating initial abstractions using abstraction to saturation and indicator-guided abstraction.

Figure 7 depicts a comparison of the two approaches for generating initial abstractions: the abstraction to saturation scheme (x axis), and the indicator-guided abstraction scheme described in Algorithm 2 (y axis). Each experiment included running our tool twice on the same benchmark (network and property), with an identical configuration except for the initial abstraction being used. The plot depicts the total time (log-scale, in seconds, with a 20-h timeout) spent by Marabou solving verification queries as part of the abstraction-refinement procedure. It shows that, in contrast to our intuition, abstraction to saturation almost always outperforms the indicator-guided approach. This is perhaps due to the fact that, although it might entail additional rounds of refinement, the abstraction to saturation approach tends to produce coarse verification queries that are easily solved by Marabou, resulting in an overall improved performance. We thus conclude that, at least in the ACAS Xu case, the abstraction to saturation approach is superior to that of indicator-guided abstraction.

This experiment also confirms that properties can indeed be proved on abstract networks that are significantly smaller than the original—i.e., despite the initial 4x increase in network size due to the preprocessing phase, the final abstract network on which our abstraction-enhanced approach could solve the query was usually substantially smaller than the original network. Specifically, among the abstraction to saturation experiments that terminated, the final network on which the property was shown to be SAT or UNSAT had an average size of 268.8 nodes, compared to the original 310—a 13% reduction. Because DNN verification becomes exponentially more difficult as the network size increases, this reduction is highly beneficial.

**Fig. 8.**
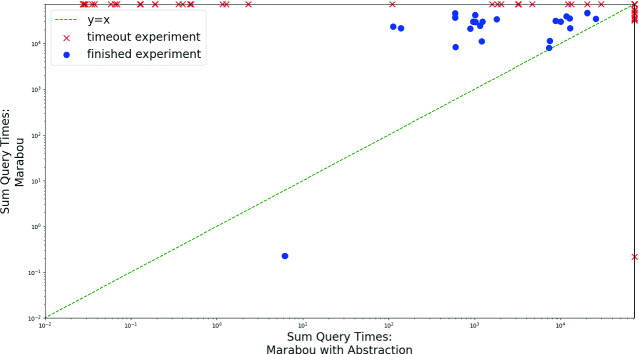
Comparing the run time (in seconds, logscale) of vanilla Marabou and the abstraction-enhanced version on the ACAS Xu benchmarks.

Next, we compared our abstraction-enhanced Marabou (in abstraction to saturation mode) to the vanilla version. The plot in Fig. 8 compares the total query solving time of vanilla Marabou (y axis) to that of our approach (x axis). We ran the tools on 90 ACAS Xu benchmarks (2 properties, checked on each of the 45 networks), with a 20-h timeout. We observe that the abstraction-enhanced version significantly outperforms vanilla Marabou on average—often solving queries orders-of-magnitude more quickly, and timing out on fewer benchmarks. Specifically, the abstraction-enhanced version solved 58 instances, versus 35 solved by Marabou. Further, over the instances solved by both tools, the abstraction-enhanced version had a total query median runtime of 1045 s, versus 63671 s for Marabou. Interestingly, the average size of the abstract networks for which our tool was able to solve the query was 385 nodes—which is an increase compared to the original 310 nodes. However, the improved runtimes demonstrate that although these networks were slightly larger, they were still much easier to verify, presumably because many of the network’s original neurons remained abstracted away.

Finally, we used our abstraction-enhanced Marabou to verify *adversarial robustness* properties [35]. Intuitively, an adversarial robustness property states that slight input perturbations cannot cause sudden spikes in the network’s output. This is desirable because such sudden spikes can lead to misclassification of inputs. Unlike the ACAS Xu domain-specific properties [20], whose formulation required input from human experts, adversarial robustness is a *universal property*, desirable for every DNN. Consequently it is easier to formulate, and has received much attention (e.g., [2, 10, 20, 36]).

In order to formulate adversarial robustness properties for the ACAS Xu networks, we randomly sampled the ACAS Xu DNNs to identify input points where the selected output advisory, indicated by an output neuron $y_i$, received a much lower score than the second-best advisory, $y_j$ (recall that the advisory with the lowest score is selected). For such an input point $x_0$, we then posed the verification query: does there exist a point *x* that is close to $x_0$, but for which $y_j$ receives a lower score than $y_i$? Or, more formally: $\left( \Vert x - x_0 \Vert _{L_\infty }\le \delta \right) \wedge (y_j \le y_i).$ If this query is SAT then there exists an input *x* whose distance to $x_0$ is at most $\delta$, but for which the network assigns a better (lower) score to advisory $y_j$ than to $y_i$. However, if this query is UNSAT, no such point *x* exists. Because we select point $x_0$ such that $y_i$ is initially much smaller than $y_j$, we expect the query to be UNSAT for small values of $\delta$.

**Fig. 9.**
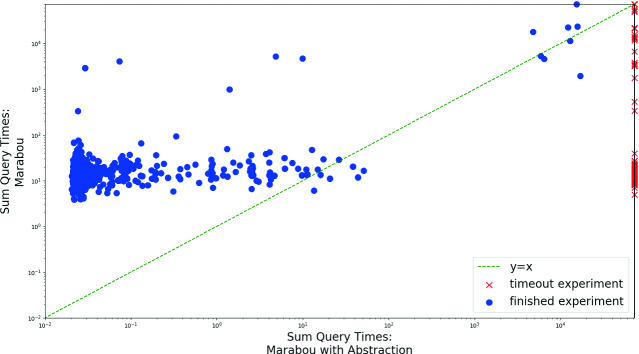
Comparing the run time (seconds, logscale) of vanilla Marabou and the abstraction-enhanced version on the ACAS Xu adversarial robustness properties.

For each of the 45 ACAS Xu networks, we created robustness queries for 20 distinct input points—producing a total of 900 verification queries (we arbitrarily set $\delta =0.1$). For each of these queries we compared the runtime of vanilla Marabou to that of our abstraction-enhanced version (with a 20-h timeout). The results are depicted in Fig. 9. Vanilla Marabou was able to solve more instances—893 out of 900, versus 805 that the abstraction-enhanced version was able to solve. However, on the vast majority of the remaining experiments, the abstraction-enhanced version was significantly faster, with a total query median runtime of only 0.026 s versus 15.07 s in the vanilla version (over the 805 benchmarks solved by both tools). This impressive 99% improvement in performance highlights the usefulness of our approach also in the context of adversarial robustness. In addition, over the solved benchmarks, the average size of the abstract networks for which our tool was able to solve the query was 104.4 nodes, versus 310 nodes in each of the original networks—a 66% reduction in size. This reinforces our statement that, in many cases, DNNs contain a great deal of unneeded neurons, which can safely be removed by the abstraction process for the purpose of verification.

## Related Work

In recent years, multiple schemes have been proposed for the verification of neural networks. These include SMT-based approaches, such as Marabou [22, 23], Reluplex [20], DLV [17] and others; approaches based on formulating the problem as a mixed integer linear programming instance (e.g., [4, 7, 8, 36]); approaches that use sophisticated symbolic interval propagation [37], or abstract interpretation [10]; and others (e.g., [1, 18, 25, 27, 30, 38, 39]). These approaches have been applied in a variety of tasks, such as measuring adversarial robustness [2, 17], neural network simplification [11], neural network modification [12], and many others (e.g., [23, 34]). Our approach can be integrated with any sound and complete solver as its engine, and then applied towards any of the aforementioned tasks. Incomplete solvers could also be used and might afford better performance, but this could result in our approach also becoming incomplete.

Some existing DNN verification techniques incorporate abstraction elements. In [31], the authors use abstraction to over-approximate the Sigmoid activation function with a collection of rectangles. If the abstract verification query they produce is UNSAT, then so is the original. When a spurious counterexample is found, an arbitrary refinement step is performed. The authors report limited scalability, tackling only networks with a few dozen neurons. Abstraction techniques also appear in the AI2 approach [10], but there it is the input property and reachable regions that are over-approximated, as opposed to the DNN itself. Combining this kind of input-focused abstraction with our network-focused abstraction is an interesting avenue for future work.

## Conclusion

With deep neural networks becoming widespread and with their forthcoming integration into safety-critical systems, there is an urgent need for scalable techniques to verify and reason about them. However, the size of these networks poses a serious challenge. Abstraction-based techniques can mitigate this difficulty, by replacing networks with smaller versions thereof to be verified, without compromising the soundness of the verification procedure. The abstraction-based approach we have proposed here can provide a significant reduction in network size, thus boosting the performance of existing verification technology.

In the future, we plan to continue this work along several axes. First, we intend to investigate refinement heuristics that can split an abstract neuron into two arbitrary sized neurons. In addition, we will investigate abstraction schemes for networks that use additional activation functions, beyond ReLUs. Finally, we plan to make our abstraction scheme parallelizable, allowing users to use multiple worker nodes to explore different combinations of abstraction and refinement steps, hopefully leading to faster convergence.
